# Generalized Developmental Enamel Hypoplasia of the Permanent Dentition Associated with Early Childhood Vitamin D Deficiency Rickets: A Case Report

**DOI:** 10.3390/dj14070399

**Published:** 2026-07-02

**Authors:** Rena Okawa, Misato Takagi, Yuto Suehiro, Kazuhiko Nakano

**Affiliations:** Department of Pediatric Dentistry, Graduate School of Dentistry, The University of Osaka, Suita 565-0871, Osaka, Japan

**Keywords:** vitamin D deficiency, rickets, enamel hypoplasia, permanent dentition, tooth development

## Abstract

**Background:** Vitamin D deficiency rickets is a metabolic bone disorder caused by impaired calcium and phosphate homeostasis resulting from insufficient vitamin D. In children, severe vitamin D deficiency can disturb the mineralization of growing bones and teeth. Although the skeletal manifestations are well recognized, reports describing generalized developmental enamel defects affecting nearly all permanent teeth remain limited. **Methods:** A 6-year-9-month-old Japanese boy with a history of vitamin D deficiency rickets diagnosed at 2 years 5 months of age was referred to our department for evaluation of generalized discoloration and morphological abnormalities affecting multiple permanent teeth. Clinical, radiographic, and medical findings were reviewed. **Results:** Laboratory examination at diagnosis revealed severe vitamin D deficiency with elevated intact parathyroid hormone levels. Possible contributing factors included exclusive breastfeeding, delayed weaning, avoidance of fish and dairy products, and limited outdoor activity. Following oral alfacalcidol supplementation, skeletal and biochemical findings gradually normalized. However, clinical examination revealed generalized enamel hypoplasia affecting the permanent incisors and first molars, characterized by yellow-brown discoloration, rough enamel surfaces, morphological irregularities, and attrition, whereas the primary dentition showed no obvious abnormalities. Panoramic radiography demonstrated generalized crown malformation involving both erupted and unerupted permanent teeth, particularly the permanent incisors, first molars, and canines, while premolars and second molars were relatively unaffected. Based on the developmental timing of the affected teeth and the patient’s medical history, the enamel defects were considered to be associated with systemic mineralization disturbance during early childhood. Restorative treatment, including composite resin restorations and stainless steel crowns, was performed to improve aesthetics and occlusal function. Preventive surgical exposure followed by composite resin restoration was also performed for the permanent canines at the onset of eruption. **Conclusions**: Severe vitamin D deficiency during critical stages of tooth development may be associated with irreversible developmental enamel defects in the permanent dentition, even after apparent systemic recovery from rickets. Early dental assessment, long-term dental follow-up, and multidisciplinary management should be considered in children with a history of nutritional rickets.

## 1. Introduction

Vitamin D is an essential hormone for maintaining calcium and phosphate homeostasis and plays a critical role in skeletal growth and mineralization [1]. Vitamin D is obtained primarily through cutaneous synthesis following sunlight exposure and, to a lesser extent, through dietary sources such as oily fish, fortified foods, and supplements [1,2]. The serum 25-hydroxyvitamin D [25(OH)D] concentration is widely accepted as the most reliable biomarker of vitamin D status [2]. According to the Global Consensus Recommendations on Prevention and Management of Nutritional Rickets, vitamin D deficiency is defined as a serum 25(OH)D concentration below 30 ng/mL [3].

Severe vitamin D deficiency during childhood causes nutritional rickets, a metabolic bone disease characterized by defective mineralization of growing bone and growth plate cartilage [3]. Clinical manifestations include bowleg deformity, delayed growth, bone pain, and skeletal fragility. Although the skeletal manifestations of rickets are well recognized, vitamin D deficiency may also affect tooth development because teeth, like bone, are highly mineralized tissues that require tightly regulated calcium and phosphate metabolism during development [1,4].

Tooth formation is a highly coordinated biological process involving reciprocal interactions between epithelial and mesenchymal tissues. Enamel formation is particularly susceptible to systemic disturbances during the secretory and maturation stages of amelogenesis. Ameloblasts require stable mineral homeostasis for normal enamel matrix formation and mineral deposition. Therefore, disturbances in calcium and phosphate metabolism caused by vitamin D deficiency may result in irreversible developmental dental defects, including enamel hypoplasia and hypomineralization [4,5]. The severity and distribution of enamel defects are thought to depend on the timing and duration of systemic disturbances during tooth development.

Recent studies have investigated associations between vitamin D deficiency during pregnancy and early childhood and developmental enamel defects in offspring, although the findings remain inconsistent [6,7,8,9,10]. Furthermore, dental manifestations such as enamel hypoplasia, delayed eruption, enlarged pulp chambers, and increased susceptibility to dental caries have been reported in children with nutritional rickets [2,11,12]. However, reports describing generalized developmental enamel defects involving nearly all permanent teeth remain limited, particularly in patients who achieved apparent systemic recovery following treatment for rickets. In addition, few reports have demonstrated a close chronological relationship between the distribution of affected teeth and the timing of untreated vitamin D deficiency or described long-term preventive dental management. Therefore, we report a case of generalized developmental enamel hypoplasia affecting nearly all permanent teeth in a child with a history of vitamin D deficiency rickets, highlighting the temporal relationship between early childhood vitamin D deficiency and permanent tooth development, as well as the importance of long-term preventive dental management.

## 2. Case Description

A 6-year-9-month-old Japanese boy was referred to the Pediatric Dentistry Clinic of Osaka University Dental Hospital by his general dentist for evaluation of abnormal color and morphology affecting multiple permanent teeth, which were suspected to be associated with a systemic disorder. According to his guardian, the primary incisors, which had already exfoliated, had exhibited normal color and smooth enamel surfaces without apparent structural abnormalities. No relevant family history of hereditary enamel disorders, metabolic bone disease, or developmental dental abnormalities was identified. The patient was born at term (38 weeks of gestation) with a birth weight of 2876 g. There was no history of neonatal illness, significant infection, prematurity, low birth weight, prolonged hospitalization, recurrent febrile illness, or prolonged antibiotic exposure during infancy.

The patient had a history of vitamin D deficiency rickets during early childhood. Bowleg deformity was first noted after 1 year of age when he began walking independently. Initially, an orthopedic surgeon considered the condition to represent physiological genu varum, and the patient was kept under observation. However, because the bowing progressively worsened over time, he was referred to the orthopedic department of a children’s hospital for further evaluation.

**Figure 1:**
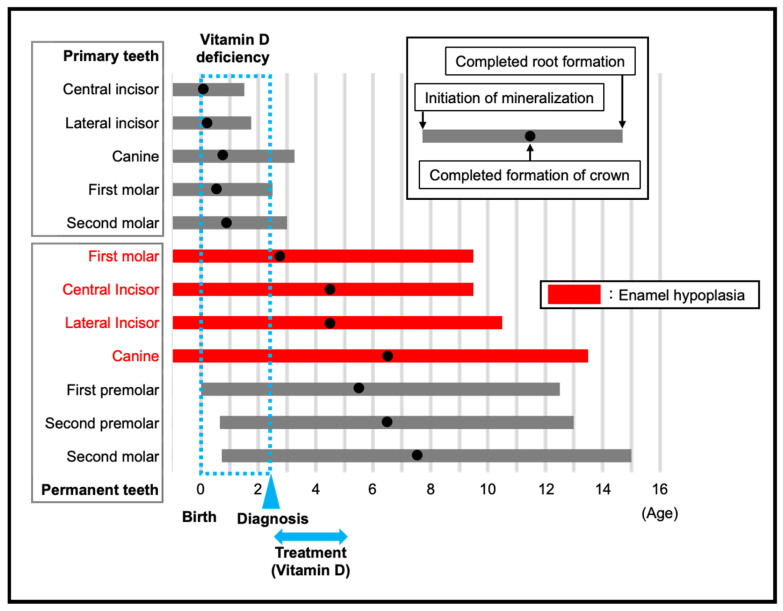
Schematic illustration of the relationship between the timing of permanent tooth mineralization and vitamin D deficiency during early childhood. The timing of tooth calcification and development was adapted from the chart of Schour and Massler [13]. The mineralization period of the permanent incisors, canines, and first molars corresponded to the period of untreated vitamin D deficiency rickets.

Radiographic examination demonstrated characteristic rachitic changes, including cupping and fraying of the distal femur and tibia, leading to a diagnosis of rickets. Laboratory examination revealed hypocalcemia (serum calcium, 6.4 mg/dL), elevated alkaline phosphatase (3162 IU/L), an elevated intact parathyroid hormone level (362 pg/mL), severe vitamin D deficiency with a serum 25(OH)D level of 7 ng/mL and a normal fibroblast growth factor 23 level (10 pg/mL). Because hypophosphatemic rickets, including X-linked hypophosphatemia, were also considered in the differential diagnosis, serum FGF23 was measured and found to be within the normal range. Serum biochemical findings were consistent with vitamin D deficiency rickets. Based on these clinical, radiographic, and laboratory findings, the patient was diagnosed with vitamin D deficiency rickets at 2 years 5 months of age (Figure 1).

Possible contributing factors included exclusive breastfeeding, delayed initiation of weaning foods until 7 months of age, avoidance of fish and dairy products, and limited outdoor activity. Oral supplementation with alfacalcidol (0.5 μg/day) was initiated after diagnosis. Following treatment, the skeletal findings gradually improved, and the radiographic abnormalities had almost completely normalized 2 years after the initiation of supplementation. The serum calcium, alkaline phosphatase, and intact parathyroid hormone levels also normalized, and urinary calcium excretion stabilized. The supplementation dose was gradually reduced and eventually discontinued at 5 years 1 month of age after systemic recovery had been confirmed.

**Figure 2:**
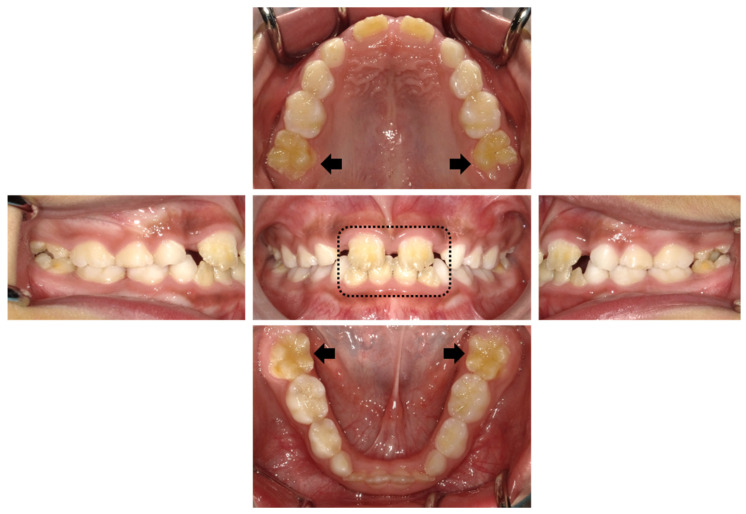
Intraoral photographs obtained on the first visit to our clinic at 6 years 9 months of age. Generalized enamel hypoplasia characterized by yellow-brown discoloration and surface irregularities was observed in the erupted permanent incisors and first molars. Arrows indicate permanent first molars affected by enamel hypoplasia. Dashed boxes indicate permanent incisors affected by enamel hypoplasia.

At the first visit to our clinic, extraoral examination revealed no obvious facial asymmetry or temporomandibular joint abnormalities. Intraoral examination demonstrated no apparent abnormalities in the remaining primary dentition. By contrast, multiple erupted permanent teeth, including the maxillary and mandibular incisors and first permanent molars, exhibited generalized enamel defects characterized by yellow-brown discoloration, rough enamel surfaces, and morphological irregularities consistent with enamel hypoplasia (Figure 2). The enamel defects were particularly severe in the maxillary central incisors and first permanent molars, where substantial enamel loss and attrition were observed. The mandibular incisors also demonstrated crown surface irregularities and discoloration, although the severity was relatively mild compared with that observed in the maxillary incisors.

Although no obvious dentin hypersensitivity was observed at the initial examination, occlusal vertical height was decreased because of enamel defects and progressive attrition. The patient and guardian primarily complained of poor dental appearance and expressed concerns regarding future tooth deterioration. Oral hygiene status was relatively good, and no extensive dental caries or pulpal symptoms were detected at the first visit.

**Figure 3:**
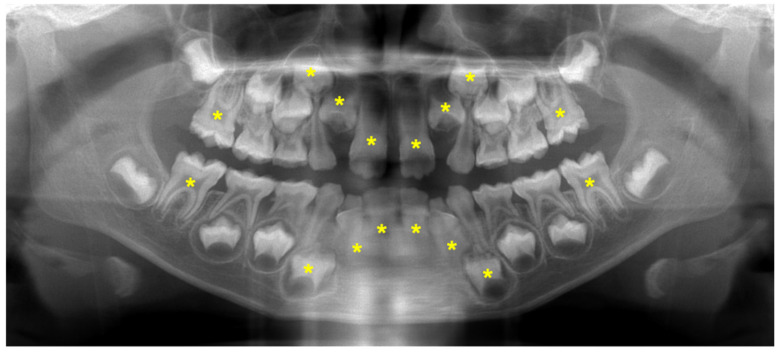
Orthopantomogram obtained at the first dental examination at 6 years 9 months of age. Generalized crown malformation and enamel defects were observed in both erupted and unerupted permanent teeth. Teeth affected by enamel hypoplasia are indicated by asterisks.

Panoramic radiography demonstrated generalized crown malformation involving erupted permanent teeth and unerupted permanent canines (Figure 3). The enamel layer appeared thin and irregular in several developing teeth. The distribution pattern of the affected teeth corresponded closely with the developmental period during which the patient experienced untreated severe vitamin D deficiency before initiation of therapy (Figure 1). By contrast, the primary dentition, most of which completes crown mineralization during the prenatal and early infancy periods, showed no apparent abnormalities. Based on the patient’s medical history, the chronological timing of tooth development, and the characteristic dental findings, the patient was diagnosed with developmental enamel hypoplasia associated with early childhood vitamin D deficiency rickets.

Because no family history suggestive of amelogenesis imperfecta was identified and the enamel defects corresponded chronologically with the period of systemic mineralization disturbance, hereditary enamel disorders were considered less likely. In addition, the generalized distribution of the defects differed from the typical clinical presentation of molar-incisor hypomineralization.

**Figure 4:**
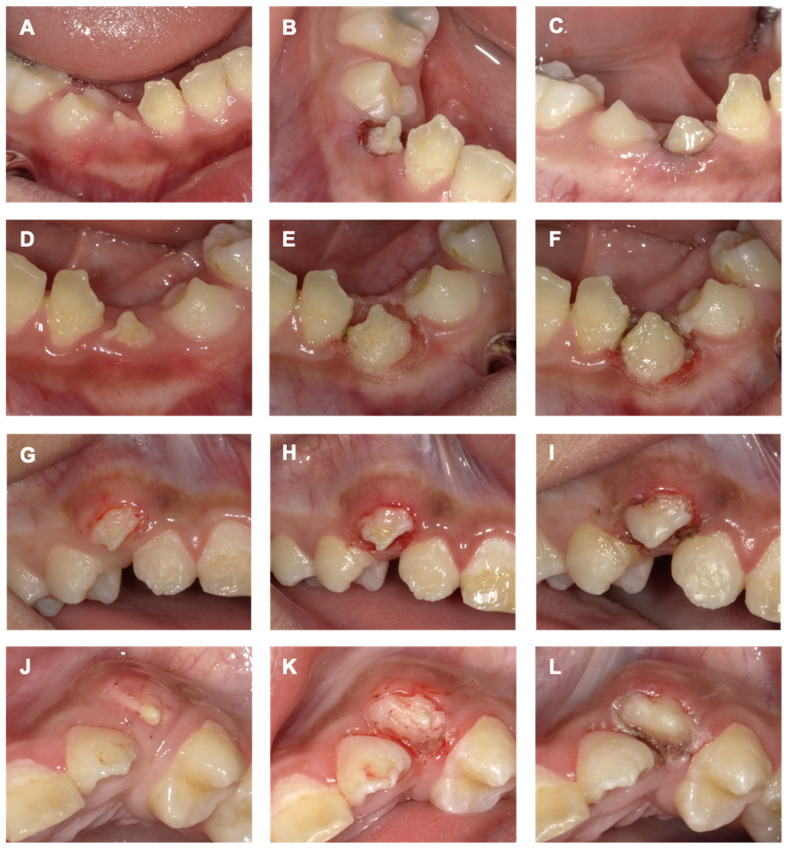
Intraoral photographs of the canines during eruption management and restorative treatment. (**A**–**C**) Mandibular right canine at 9 years 4 months of age. (**D**–**F**) Mandibular left canine at 9 years 10 months of age. (**G**–**I**) Maxillary right canine at 11 years 2 months of age. (**J**–**L**) Maxillary left canine at 11 years 5 months of age. (**A**, **D**, **G**, **J**) Before surgical exposure. (**B**, **E**, **H**, **K**) After surgical exposure. (**C**, **F**, **I**, **L**) After composite resin restoration.

**Figure 5:**
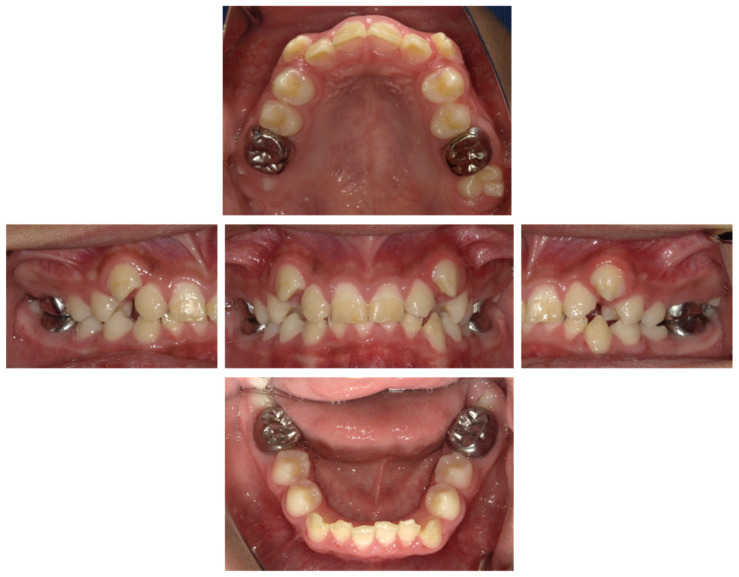
Intraoral photographs obtained after restorative treatment at 12 years 5 months of age. Composite resin restorations and stainless steel crowns were placed to restore aesthetics and occlusal function in the hypoplastic permanent teeth.

For aesthetic rehabilitation, composite resin restorations were placed on the labial surfaces of the maxillary central incisors. Stainless steel crowns were applied to the maxillary and mandibular first permanent molars as temporary prosthetic restorations after complete eruption to restore occlusal vertical dimension and prevent further enamel breakdown. In addition, because all permanent canines were expected to exhibit morphological abnormalities associated with enamel hypoplasia, surgical exposure was performed immediately after eruption began, followed by composite resin restoration to prevent enamel fracture (Figure 4). No obvious enamel hypoplasia was observed in any of the premolars or second molars (Figure 5). Periodic follow-up and preventive management, including fluoride application and oral hygiene instruction, were continued. Definitive prosthetic rehabilitation was planned after completion of the permanent dentition and craniofacial growth.

## 3. Discussion

Vitamin D deficiency during childhood can affect not only skeletal development but also tooth formation and mineralization. In the present case, severe vitamin D deficiency during early childhood was associated with generalized developmental enamel hypoplasia affecting multiple permanent teeth. The patient exhibited characteristic enamel defects in the permanent incisors and first molars, whereas the primary dentition showed no obvious abnormalities. This difference may be explained by the timing of tooth development and mineralization.

Tooth development is a highly regulated biological process involving reciprocal interactions between epithelial and mesenchymal tissues. Ameloblasts are particularly sensitive to systemic disturbances during enamel formation because enamel, unlike bone, cannot undergo remodeling after completion of mineralization. Vitamin D plays an important role in calcium and phosphate homeostasis, both of which are essential for normal enamel matrix formation and mineral deposition [1,4]. Severe vitamin D deficiency may therefore impair ameloblast function and disturb enamel mineralization, leading to irreversible developmental defects such as enamel hypoplasia and hypomineralization [4,5]. Experimental and clinical studies have suggested that systemic disturbances during the secretory and maturation stages of amelogenesis can alter enamel thickness, mineral density, and surface morphology. In severe cases, prolonged disturbances may result in generalized developmental defects affecting multiple teeth.

The chronology of permanent tooth development in the present case closely corresponded with the period of untreated severe vitamin D deficiency during early childhood. Permanent incisors, first molars, and canines, which undergo active mineralization during infancy and early childhood, exhibited generalized enamel defects and crown malformation, whereas premolars and second molars showed no obvious abnormalities. By contrast, the primary dentition, most of which completes crown mineralization during the prenatal and early infancy periods, appeared clinically unaffected. These findings suggest that the timing and duration of systemic mineralization disturbance selectively affected developing permanent teeth according to their stage of mineralization and may represent critical determinants of developmental dental defects.

Consistent with this observation, previous studies have reported associations between prenatal and early childhood vitamin D deficiency and developmental enamel defects in both primary and permanent dentitions [6,7,8,9,10]. Furthermore, several reports have described dental manifestations in children with nutritional rickets, including enamel hypoplasia, delayed eruption, enlarged pulp chambers, and increased susceptibility to dental caries [2,11,12]. Recent systematic reviews have also suggested a possible association between vitamin D deficiency and developmental enamel defects, although the current evidence remains limited and further well-designed studies are required to clarify the nature of this relationship [14].

Differential diagnosis should also be considered when generalized enamel defects are observed. Amelogenesis imperfecta is a hereditary disorder characterized by generalized enamel abnormalities involving both primary and permanent dentitions. However, our patient had no family history suggestive of hereditary enamel disorders, and the primary dentition showed no clinically apparent abnormalities. Moreover, the chronological distribution of the affected permanent teeth corresponded closely with the period of severe untreated vitamin D deficiency, supporting an acquired developmental etiology rather than a genetic disorder.

Molar-incisor hypomineralization was also considered in the differential diagnosis because the permanent first molars and incisors were severely affected. However, molar-incisor hypomineralization typically presents as demarcated opacities with qualitative enamel defects, whereas the present case demonstrated generalized enamel surface irregularities, crown malformation, and widespread involvement of nearly all permanent teeth, including unerupted teeth. These findings were more consistent with generalized developmental enamel hypoplasia associated with systemic mineralization disturbance.

Although enamel hypoplasia was the predominant finding in the present case, hypoplastic and hypomineralized changes may coexist in patients with severe systemic disturbances during tooth development. Clinically, the affected teeth demonstrated yellow-brown discoloration, rough enamel surfaces, and enamel fragility, suggesting that impaired mineralization may also have contributed to the observed dental phenotype.

An important clinical implication of this case is that dental complications may become apparent several years after apparent recovery from rickets. Pediatricians, orthopedic surgeons, pediatric dentists, and general dentists should therefore recognize that children with severe vitamin D deficiency require long-term dental follow-up even after normalization of skeletal and biochemical findings. These findings are consistent with recent evidence demonstrating that pediatric bone diseases are frequently associated with dentomaxillofacial abnormalities and emphasize the importance of interdisciplinary collaboration and long-term dental follow-up in affected children [15]. Early dental referral may facilitate preventive intervention before severe enamel breakdown and occlusal deterioration occur.

From a dental management perspective, preservation of tooth structure and maintenance of occlusal function are particularly important because hypoplastic enamel is highly susceptible to attrition and fracture. In the present case, stainless steel crowns were used as temporary restorations for the first permanent molars to restore occlusal vertical dimension and prevent further enamel loss during growth. Composite resin restorations were selected for the anterior teeth to improve aesthetics and maintain oral function. Furthermore, because morphological abnormalities of the permanent canines were anticipated based on panoramic radiographic findings, the patient’s guardian was instructed to visit our clinic at the onset of canine eruption. Timely surgical exposure followed by composite resin restoration successfully prevented enamel fracture and further structural breakdown of the affected teeth. Preventive management, including oral hygiene instruction, fluoride application, and periodic monitoring, was also considered important because structurally compromised enamel may increase the risk of future dental complications. At the most recent follow-up, the restorations remained functional, and both the patient and guardian were satisfied with the treatment outcomes.

Long-term multidisciplinary management is necessary in patients with severe developmental enamel defects. Because craniofacial growth and eruption of the remaining permanent teeth were incomplete in the present patient, definitive prosthetic rehabilitation was postponed until completion of growth. Continuous follow-up is required to monitor occlusal development, eruption of unerupted teeth, restorative integrity, and future prosthodontic needs.

This report has several limitations. First, it describes a single patient, and therefore, the findings may not be generalizable to all children with vitamin D deficiency rickets. Second, genetic testing was not performed; therefore, hereditary conditions associated with developmental enamel defects, including amelogenesis imperfecta caused by de novo variants, cannot be completely excluded. Third, dietary history was obtained retrospectively from the patient’s guardian and may be subject to recall bias. Finally, the exact onset and duration of vitamin D deficiency before diagnosis could not be determined because maternal and neonatal vitamin D status were unavailable. Therefore, although the distribution of affected teeth appeared to correspond with the period of untreated vitamin D deficiency, the possibility of earlier deficiency cannot be excluded.

This case highlights the importance of adequate nutritional management and sunlight exposure during early childhood for normal skeletal and dental development. Early recognition and treatment of vitamin D deficiency are important not only for preventing skeletal complications but also for preserving normal permanent tooth development. Clinicians should be aware that irreversible developmental dental defects may become evident several years after apparent systemic recovery from nutritional rickets.

## 4. Conclusions

In conclusion, children with vitamin D deficiency rickets may develop irreversible developmental defects in the permanent dentition even after apparent systemic recovery. Early dental assessment and long-term dental follow-up should therefore be considered in children with severe vitamin D deficiency during early childhood. Multidisciplinary management may help minimize future oral complications and preserve oral function.

## Data Availability

The original contributions presented in this study are included in the article. Further inquiries can be directed to the corresponding author.

## Abbreviation

The following abbreviation is used in this manuscript:

25(OH)D	25-hydroxyvitamin D
